# Immunoglobulin G promotes skin graft acceptance in an immunologically potent rat model

**DOI:** 10.18632/oncotarget.9823

**Published:** 2016-06-05

**Authors:** Xingmu Liu, Tao Huang, Xueling Chen, Meiling Yan, Feiyuan Yu, Huan Gu, Chao He, Jiang Gu

**Affiliations:** ^1^ Department of Pathology and Provincial Key Laboratory of Infectious Diseases and Immunopathology, Collaborative and Creative Center, Molecular Diagnosis and Personalized Medicine, Shantou University Medical College, Shantou, Guangdong, China; ^2^ Department of General Surgery, Second Affiliated Hospital, Shantou University Medical College, Shantou, Guangdong, China

**Keywords:** immunoglobulin G, rat model, transplantation, rejection, cytokines, Immunology and Microbiology Section, Immune response, Immunity

## Abstract

Immunoglobulin G (IgG) has been shown to protect graft rejection after transplantation, whereas the molecular mechanism of IgG in promoting graft acceptance has not been well established. In this study, we tested the effectiveness of IgG in preventing rejection of transplanted skin graft in an immunologically potent rat model, and studied the mechanism of this protection. We found that systemic or local administration of IgG significantly prolonged the survival of skin grafts with the immune tolerance induced by IgG and subcutaneous local injection of 1mg IgG to adult SD rat yielded the longest survival of skin grafts from 5.8 to 17.3 days. We also found that IgG reduced the number of pro-inflammatory cells especially lymphocytes, neutrophils and basophils, increased the seral levels of anti-inflammatory factors including IL-10 and IL-4, and activated CD4^+^CD25^+^Foxp3^+^ regulatory T cells, unveiling the mechanisms of this protective effect. These findings provide new insight to support clinical application of IgG in treating transplantation.

## INTRODUCTION

In 1952, IgG was first used to treat immunodeficiency disease by Ogden Bruton [1]. Intravenous immunoglobulin (IVIG) which is prepared from the plasma of a thousand or more blood donors was initially shown to be effective in treating acute idiopathic thrombocytopenic purpura (ITP) in 1981 [2]. From then on, IVIG has been used to treat different kinds of illnesses including immunodeficiency [3], autoimmune and inflammatory diseases [4], neurologic diseases [5], severe autoimmune blistering diseases [6] etc with little side effects.

IgG has been shown to decrease the severity of acute graft-*versus*-host disease (GVHD) after clinical bone marrow transplantation and in an experimental setting [7, 8]. IgG was also shown to have protective effect against acute rejection of kidney, heart and liver transplantations [9–16]. However, the molecular mechanism of IgG in promoting graft acceptance has not been well understood. In addition, it has been unable to prolong allogeneic skin graft survival with IVIG treatment in immunologically potent mice [17].

In the present study, we transplanted skin grafts between immunologically potent wild-type rats and injected recipient rats with IgG in different dosages *via* different administration approach. The survival durations of the skin grafts were examined. The results showed that injection of IgG significantly prolonged the survival duration of skin graft, and subcutaneous injection of IgG achieved the longest graft tolerance. Factors that mediated this immune tolerance were also investigated. This study elucidates the mechanism of IgG induced graft tolerance and provides evidence to support clinical application of IgG in treating transplantation rejection.

## RESULTS

### IgG promotes skin graft acceptance in a dose-dependent manner

Skin graft rejection began at day 3 or 4 after skin transplantation without IgG treatment. The effectiveness of IgG in preventing rejection was shown in Figure 2 and Figure 3. Injection of 0.1mg and 5mg IgG had no significant effect on skin graft acceptance in comparison to the PBS control group no matter through tail vein or subcutaneous injection. The rejections were complete before or at day 7 after transplantation. Injection of 0.5mg IgG through tail vein also has no significant protecting effect. Injection of 2mg IgG through tail vein and 0.5mg or 2mg IgG subcutaneously showed weak protecting effect, delaying complete rejection to day 10 or later after transplantation. Injection of 1mg IgG yielded the longest survival of skin graft to day 12 or longer after transplantation. Therefore, we chose 1mg IgG as the injection dosage for subsequent experiments.

**Figure 1:**
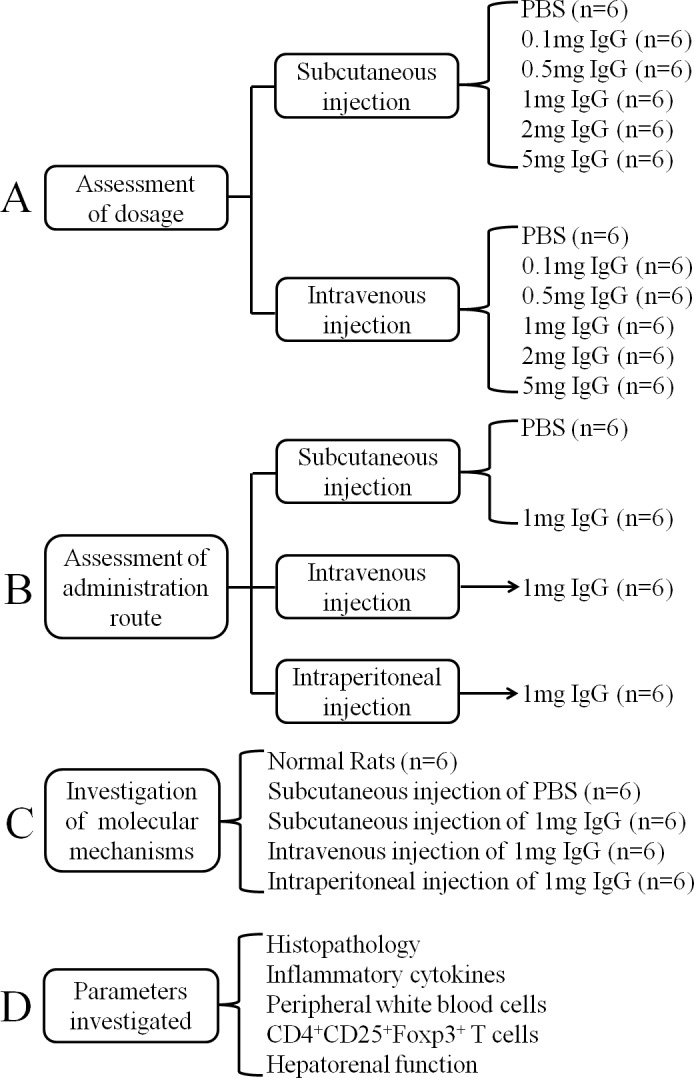
A diagram shows the experimental design of various groups of the rat skin transplant model **A.** Groups of rats uesd to assess the effect of different dosages of IgG injection in promoting allograft tolerance. **B.** Groups of rats used to assess the effect of different administration routes of IgG injection in promoting allograft tolerance. **C.** Groups of rat used to investigate the molecular mechanisms of IgG in promoting allograft tolerance. **D.** Parameters examined to investigate the molecular mechanisms of IgG in promoting allograft tolerance.

**Figure 2:**
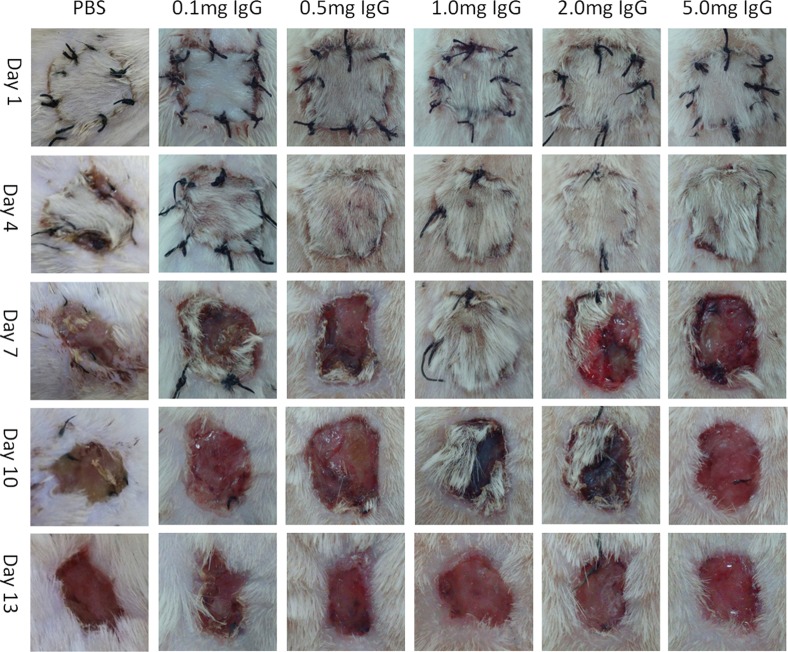
Graft survival of intravenous injection with different dosages of IgG At day 1 after transplantation, different dosages of IgG including 0.1mg, 0.5mg, 1mg, 2mg or 5mg were injected into each rat through tail vein. Injection of PBS through tail vein was used as a control. Graft rejection of PBS, 0.1mg, 0.5mg and 5mg Iv-Inj groups were all completed before or at day 7 after surgery. The 2mg Iv-Inj group showed severe rejection at day 7 and the rejection was completed at day 13. The 1mg Iv-Inj group showed no rejection at day 7 and complete rejection at day 13 after transplantation.

**Figure 3:**
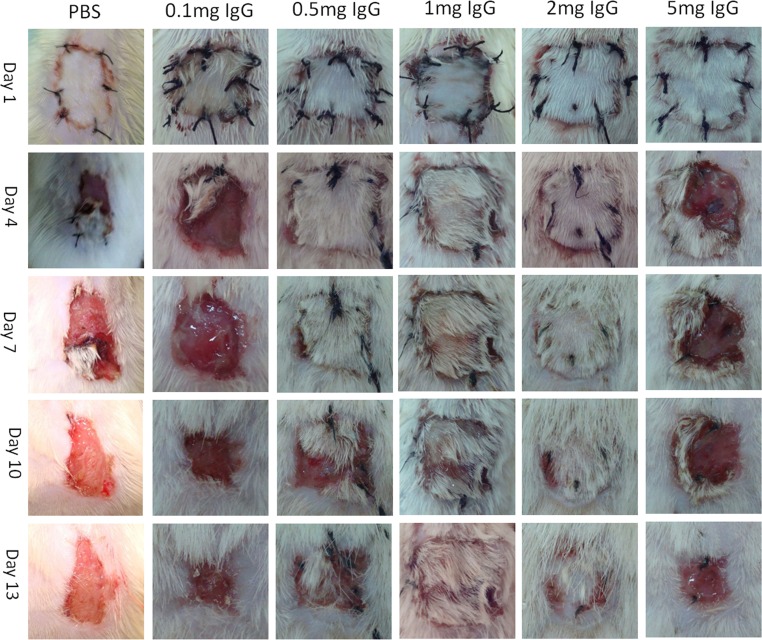
Graft survival of subcutaneous injection with different dosages of IgG At day 1 after transplantation, different dosages of IgG including 0.1mg, 0.5mg, 1mg, 2mg or 5mg were subcutaneously injected into recipient rats. Subcutaneous injection of PBS was used as a control. Graft rejection of PBS, 0.1mg and 5mg Sub-Inj groups were all completed before or at day 7 after surgery. The 0.5mg Sub-Inj group began to reject at day 7 and completed at day 13. The 2mg Sub-Inj group began to reject at day 10 and completed at day 13. The 1mg Sub-Inj group showed almost no rejection at day 13 after transplantation, and the rejection was not completed until day 17.

### Subcutaneous injection of 1mg IgG (Sub-Inj) showed the most effective protection for skin graft acceptance

**Figure 4:**
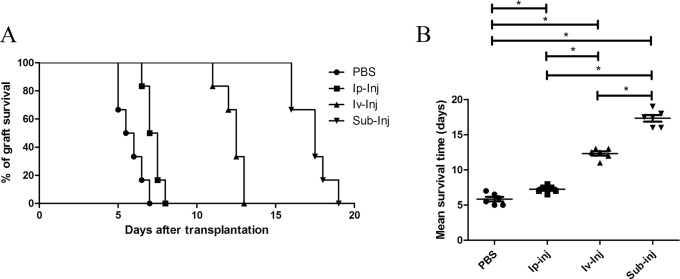
The percent of graft survival at days after transplantation and mean survival durations of grafts with different injection approaches At day 1 after transplantation, 1mg IgG was injected into recipient rats with different routes including intraperitoneal, intravenous and subcutaneous. Subcutaneous injection of PBS was used as a control. **A.** The percent of graft survival at days after transplantation. **B.** Mean graft survival days with different methods. *P < 0.05 indicates statistical significance, *n* = 6.

1mg IgG (each rat) was injected into recipient rats through different administration routes including intraperitoneal injection, subcutaneous injection and intravenous injection. Subcutaneous injection of PBS was used as a control. As shown in Figure 4A & 4B, injection of IgG through 3 different routes all prolonged the survival duration of the skin grafts, and Sub-Inj induced showed the longest duration of graft tolerance. The survival durations of the transplanted skin grafts in subcutaneous PBS injection group, intraperitoneal injection of 1mg IgG (Ip-Inj) group, intravenous injection of 1mg IgG (Iv-Inj) group and Sub-Inj group were (5.8 ± 0.3), (7.3 ± 0.2), (12.3 ± 0.3) and (17.3 ± 0.5) days respectively.

### Pathology of the transplanted skin graft

**Figure 5:**
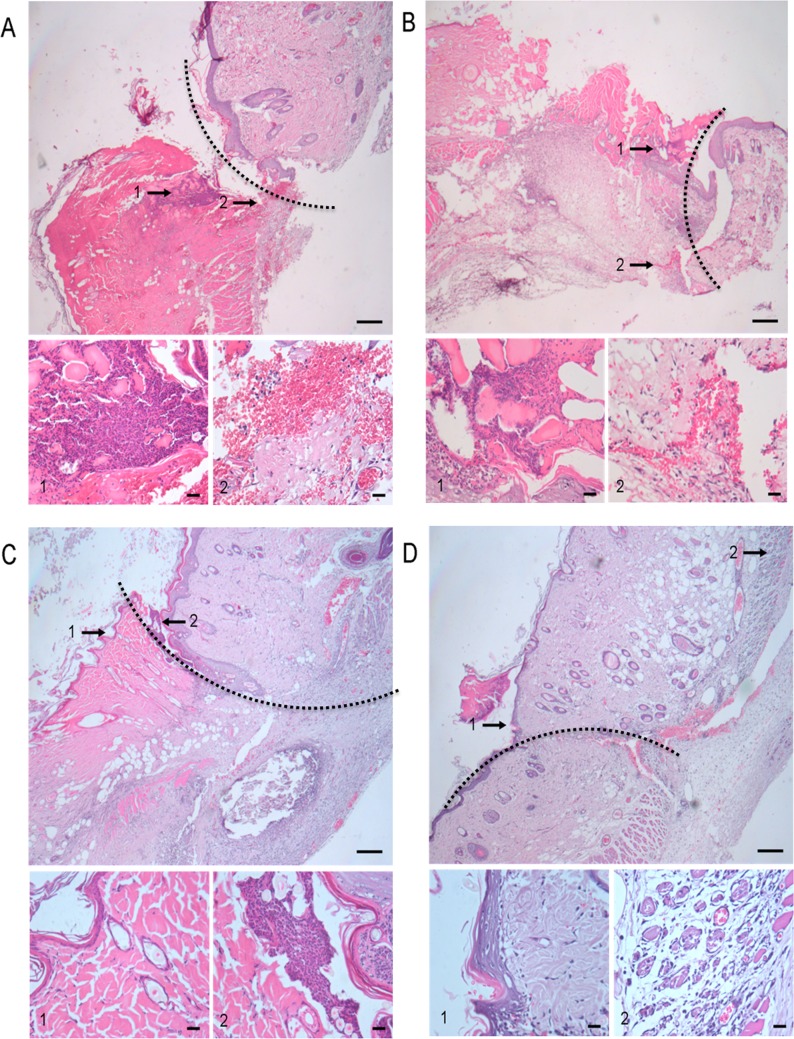
Typical pathological appearance of different groups at day 4 after transplantation At day 4 after transplantation, the pathological changes of skin graft and the adjacent host skin were examined with H&E staining. (**A.**-**D.** respectively represent the typical pathological appearance of PBS, 1mg Ip-Inj, 1mg Iv-Inj and 1mg Sub-Inj groups. Insets1 and 2 show higher magnification of corresponding areas. The internal and outside of curves respectively showed adjacent host skin and skin graft portions. Scale bars, 20 μm.

At day 4 after transplantation, the pathology of skin graft and adjacent host skin was examined with H&E staining. As shown in Figure 5A, the skin graft of control (PBS group) showed the most intense rejection and the worst tissue morphology with leukocyte infiltration, dermis edema, hemorrhage, partial tissue necrosis, vasculitis, folliculitis and epidermis separation. Typical vasculitis and folliculitis were much milder in the Ip-Inj group (Figure 5B). Only a slight leukocyte infiltration and no typical vasculitis or folliculitis appeared in the Iv-Inj group (Figure 5C). Sub-Inj group showed the best tissue morphology with almost no leukocyte infiltration, vasculitis or folliculitis (Figure 5D).

### Injection of IgG significantly increased anti-inflammatory factors IL-4 and IL-10 in host serum

**Figure 6:**
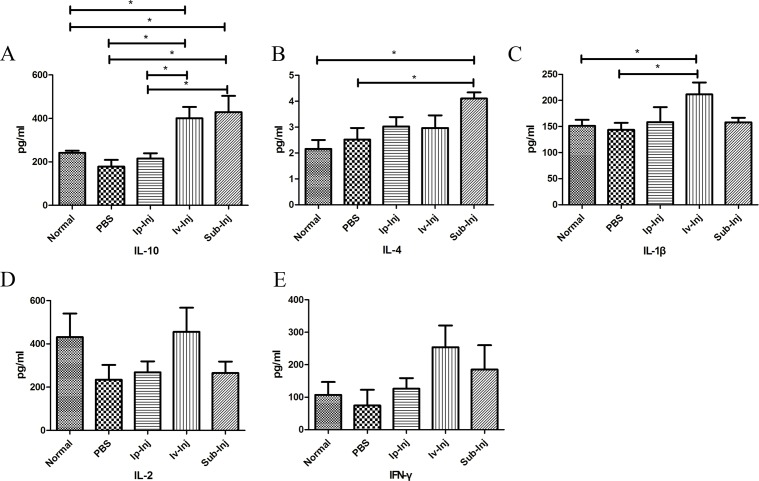
Seral levels of inflammatory factors and anti-inflammatory factors examined with ELISA At day 4 after transplantation, differences in seral anti-inflammatory factors IL-4 and IL-10 and inflammatory factors IL-1β, IL-2 and IFN-γ among normal, PBS, 1mg Ip-Inj, 1mg Iv-Inj, and 1mg Sub-Inj groups were examined with ELISA. The histogram show the levels of IL-10 **A.**, IL-4 **B.**, IL-1β **C.**, IL-2 **D.** and IFN-γ **E.** among different groups. *P < 0.05 indicates statistical significance, *n* = 6.

As shown in Figure 6A & 6B, at day 4 after transplantation, the levels of IL-10 and IL-4 of the 1mg Sub-Inj group were significantly higher than that of the normal and PBS groups. The level of anti-inflammatory factor IL-10 of 1mg Iv-Inj group was higher than that of the normal and the PBS groups (Figure 6A). The level of inflammatory factor IL-1β of 1mg Iv-Inj group was also increased (Figure 6C). There was no significant difference in the levels of IL-2 and IFN-γ among the different groups (Figure 6D & 6E).

### IgG significantly reduced the numbers of PWBC after transplantation, especially for lymphocytes, neutrophils and basophils

**Figure 7:**
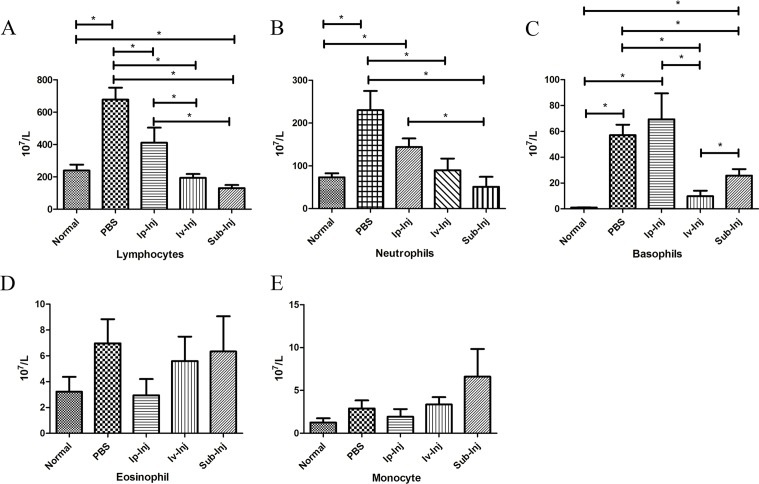
Injection of IgG significantly reduced numbers of PWBC after transplantation At day 4 after transplantation, the numbers of PWBC of different groups were analyzed with an automatic blood cell analyzer. The histograms show the numbers of lymphocyte **A.**, neutrophil **B.**, basophil **C.**, eosinophil **D.** and monocyte **E.** in the blood in normal, PBS, 1mg Ip-Inj, 1mg Iv-Inj, and 1mg Sub-Inj groups respectively. *P < 0.05 indicates statistical significance, *n* = 6.

As shown in Figure 7, transplantation significantly increased the numbers of PWBC in the serum in comparison to the normal group including lymphocytes, neutrophils and basophils. The numbers of lymphocyte, neutrophil and basophils in both the 1mg Iv-Inj and the 1mg Sub-Inj groups were significantly lower than that in the PBS group (Figure 7A, 7B & 7C). There was no statistical difference in numbers of eosinophils and monocytes among the groups (Figure 7D & 7E).

### Injection of IgG increased the numbers of CD4^+^ CD25^+^ Foxp3^+^ regulatory T cells

**Figure 8:**
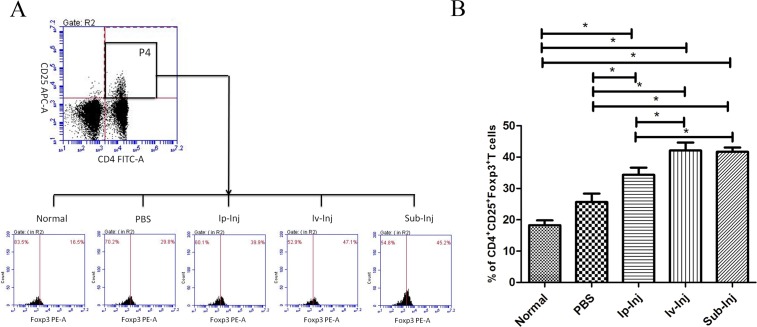
Injection of IgG increased the number of CD4^**+**^ CD25^**+**^ Foxp3^**+**^ regulatory T cells At day 4 after transplantation, the levels of CD4^+^ CD25^+^ Foxp3^+^ regulatory T cells in host blood in normal, PBS, 1mg Ip-Inj, 1mg Iv-Inj, and 1mg Sub-Inj groups were examined with flow cytometry. **A.** is a representative result. **B.** shows statistical data of six independent experiments. *P < 0.05 indicates statistical significance, *n* = 6.

At day 4 after transplantation, the level of CD4^+^ CD25^+^ Foxp3^+^ regulatory T cells in the host blood was examined with flow cytometry. As shown in Figure 8, the percentages of CD4^+^ CD25^+^ Foxp3^+^ T cells in CD4^+^ CD25^+^ regulatory T cells in 1mg Ip-Inj, 1mg Iv-Inj and 1mg Sub-Inj groups were all significantly higher than that of the PBS group.

### Injection of IgG reduced the hepatorenal lesion of transplantation

**Figure 9:**
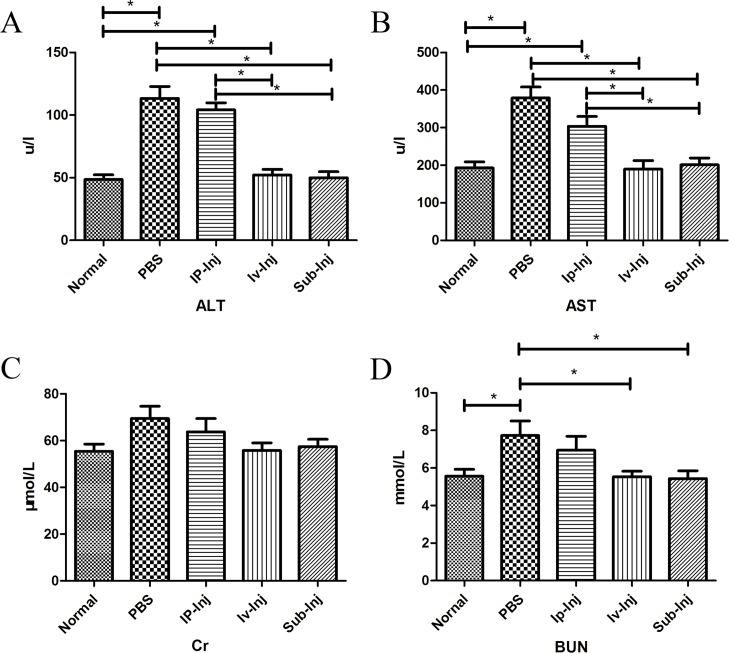
Injection of IgG reduced hepatorenal damage following transplantation At day 4 after transplantation, the levels of ALT **A.**, AST **B.**, Cr **C.** and BUN **D.** among normal, PBS, 1mg Ip-Inj, 1mg Iv-Inj, and 1mg Sub-Inj groups were examined to assess the hepatorenal damage of each group. IgG administration appears to have a protective effect on the liver and the kidneys of the recipients. *P < 0.05 indicates statistical significance, *n* = 6.

The levels of ALT, AST, Cr and BUN in the host serum were examined at day 4 after transplantation to assess the hepatorenal function. As shown in Figure 9A & 9B, liver damage caused by transplantation was revealed by the increase of ALT and AST levels. The injection of IgG through intravenous and subcutaneous routes significantly reduced liver damage with decreased ALT and AST. Likewise, administration of IgG through intravenous and subcutaneous injections also significantly reduced kidney damage with marked BUN reduction (Figure 9D). There was no significant difference in the levels of Cr among the groups (Figure 9C).

## DISCUSSION

IgG is the main component of seral immunoglobulins [18], and has been used in a variety of blistering skin diseases such as bullous pemphigus and pemphigoid [18]. It has also been used in lung, kidney, heart and liver transplantations with benefical effects [13, 16, 19, 20]. For skin transplantation, it has been reported that IVIG promoted skin allograft acceptance by triggering functional activation of CD4^+^ Foxp3^+^ T cells, but it failed to demonstrate that IVIG treatment could prolong allogeneic skin allograft survival in immunologically potent wild-type mice [17]. Our study demonstrated the marked effectiveness of IgG in promoting skin graft acceptance in an immunologically potent wild-type rat model, and showed that this protective effect was dose- and route- dependent.

The optimal dosage of IgG in clinical application in treating various illnesses has been controversial [21]. Our results showed that dosage is important for IgG administration. We found that injection of 1mg IgG in a 250 ±10g adult immunologically intact wild-type SD rat model yielded the most effective result in protecting the skin grafts. Comparing with this dosage, lower or higher dosages were less effective in protecting the skin graft.

Since the first report of IgG injection in treating primary immunodeficiency (PID) [1], intravenous injection of IgG has been the most commonly used approach of administration. Nevertheless, due to severe and/or systemic adverse reactions and the difficulties in gaining venous access, subcutaneous injection of IgG was introduced as an alternative route in Europe and the USA only a few years ago [22, 23]. In the present study, we found that the survival duration of skin grafts varied among different administration approaches (Figure 4); Sub-Inj IgG was the most effective when compared to the other approaches. At day 4 after transplantation, pathology examination found that the Sub-Inj group had few leukocyte infiltration, and no vasculitis or folliculitis. The structure of corium layer was clear without edema, bleeding or necrosis (Figure 5). Taken together, subcutaneous injection of IgG appeared to be the most effective approach in treating skin graft.

Lymphocyte infiltration is known to be a characteristic feature of skin graft rejection [24]. In this study, treatment with IgG was found to reduce lymphocyte infiltration (Figure 5) and the numbers of peripheral blood lymphocytes, neutrophils and basophils (Figure 7). In previous studies, it was noted that IgG suppressed the proliferation of T cells [25] and differentiation and maturation of dendritic cells [26]. It is likely that suppression of cellular immunity might be the strategy for IgG to protect the skin graft from rejection, but the mechanisms by which IgG attenuates cellular immunity need further investigation.

Cytokines are known to be involved in graft rejection and tolerance. Rejection is generally accompanied by increase of Th1 cytokines (IL-2 and IFN-γ) and decrease of Th2 cytokines (IL-10 and IL-4). In tolerance models, however, the expression pattern was reversed with lower expression of Th1 cytokines (IL-2 and IFN-γ) and higher expression of Th2 cytokines (IL-10 and IL-4) [27]. In this study, we found that injection IgG significantly increased the levels of anti-inflammatory factors IL-4 and IL-10 in the recipient rats (as shown in Figure 6A & 6B), indicating that increasing the levels of anti-inflammatory factors may also be a mechanism for IgG to promote skin graft acceptance. The higher level of inflammatory factor IL-1β in Iv-Inj group than that in Sub-Inj group may partly explain the less effectiveness of the former than the later (Figure 6C).

It has been shown that CD4^+^ CD25^+^ regulatory T cells played a key role in the rejection of transplanted allograft [28, 29]. These subgroups of T cells can restrain the activation and proliferation of other immunologic effector cells. It has also been reported that CD4^+^CD25^+^ regulatory T cells may influence the functions of other immune cells including macrophages, dendritic cells and NK cells [30, 31]. Foxp3 was essential for the growth and functional development of CD4^+^CD25^+^ regulatory T cells [32]. Therefore, CD4^+^CD25^+^Foxp3^+^ is a marker of regulatory T cells [33–37]. In this study, the percentages of CD4^+^ CD25^+^ Foxp3^+^ T cells in CD4^+^ CD25^+^ regulatory T cells of 1mg Ip-Inj, 1mg Iv-Inj and 1mg Sub-Inj groups were all significantly higher than that of the PBS control group (Figure 8). This result indicates that the potential effect of IgG in prolonging the survival of the skin allograft might be achieved through increasing the percentage of CD4^+^CD25^+^Foxp3^+^ regulatory T cells.

We also found that skin allograft transplantation significantly damaged hepatorenal function as ALT, AST and BUN levels were all markedly increased (Figure 9). The injection of IgG intravenously and subcutaneously significantly reduced blood ALT, AST and BUN levels indicating less liver and kidney damages. Therefore administration of IgG was able to promote allograft tolerance and at the same time induce few side effects.

In conclusion, we found that administration of IgG could promote skin graft acceptance, and this effect was dose- and route- dependent. Subcutaneous injection achieved the best result in comparison to systemic or peritoneal injection. In addition, we found that this protective effect might be achieved by affecting the immune responses including reducing the number of pro-inflammatory cells, especially lymphocytes, neutrophils and basophils, increasing the level of anti-inflammatory factors such as IL-10 and IL-4, and activating CD4^+^CD25^+^Foxp3^+^ regulatory T cells. These findings provide a new insight into the mechanism of IgG induced immune tolerance in skin graft transplant and support for clinical application of IgG in treating graft rejection.

## MATERIALS AND METHODS

### Animals

Adult Sprague Dawley (SD) rats were obtained from Vital River Laboratories (Beijing, China), and housed in the Animal Laboratory Centre of Shantou University Medical College. Animal experiments were performed in accordance with the Guide for the Care and Use of Laboratory Animals of Shantou University. The protocol was approved by the Committee of the Ethics of Animal Experiments of the Shantou University Medical College. SD rats aged between 8 to 10 weeks and weighing 250 ±10g were used in all experiments.

### Establishment of an immunologically potent wild-type rat skin transplant model

SD rats received intraperitoneal anesthesia with 1% pentobarbital (30 mg/kg) before transplantation. After shaving the hair of the back locally and disinfecting the area with 75% alcohol, a 1.5cm × 1.5cm full thickness back skin was transplanted between pairs of rats. At day 1 after transplantation, injection of PBS or IgG was performed. The experimental design is illustrated in Figure 1.

### Isolation of IgG from the rat serum

Total IgG was purified from the serum of SD rats with Protein G Agarose (Beyotime, Jiangsu, China) according to the manufacturer's instructions. The concentrations of IgG were measured with Pierce BCA Protein Assay Kit (Thermo Fisher Scientific Inc., Waltham, MA, USA).

### H&E staining

At day 4 after transplantation, the skin graft along with adjacent host skin were dissected and fixed in 4% paraformaldehyde, embedded in paraffin, sectioned at 4 μm and stained with H&E. The slides were photographed with a light microscope (Leica, Wetzlar, Germany).

### ELISA

The seral levels of cytokines including IL-10, IL-4, IL-1β, IL-2 and INF-γ of different groups at day 4 after transplantation were measured with an ELISA Kit (eBioscience, San Diego, CA, USA) according to the manufacturer's instructions.

### Examination of the levels of peripheral white blood cells (PWBC)

The numbers of PWBC were analyzed with a fully automatic blood cell analyzer (Boule Medical AB, Stockholm, Sweden). The reagents including CA620-Vet supporting special diluents and hemolytic agent were purchased from the same Boule Medical AB Company.

### Assessment of hepatorenal damages

In order to check whether there were side effects of IgG injection, hepatorenal function was evaluated with the examination of levels of Glutamic-pyruvic transaminase (ALT), aspartate aminotransferase (AST), urea nitrogen (BUN) and creatinine (Cr). ALT and AST were examined with the International Federation of Clinical Chemistry and laboratory medicine (IFCC) recommended method, and BUN and Cr were examined with picric acid colorimetric method. Briefly, the blood of different groups was obtained at day 4 after transplantation. The sera were separated and then examined with a Hitachi automatic biochemical analyzer 7060 c (Hitachi, Ltd. Tokyo, Japan) employing a kit provided by Beijing Nine Strong Biological Technology Co. Ltd. (Beijing, China).

### Flow cytometry

Heparinized whole blood of different groups was collected at day 4 after transplantation and aliquoted into 100 μL. The samples were first incubated with anti-surface antigens primary antibodies including FITC-conjugated anti-Rat CD4 and APC-conjugated anti-Rat CD25 (all purchased from eBioscience, San Diego, CA, USA) and then incubated with anti-intracellular (nuclear) antigen primary antibody, i.e. PE-conjugated anti-Rat Foxp3 monoclonal antibody according to the manufacturer's protocol. After removing RBCs with red blood cell lysis buffer, the samples were examined with the BD Accuri^TM^ C6 flow cytometer (Becton Dickinson, Franklin Lakes, NJ, USA).

### Statistical analysis

We used statistical software Prism (GraphPad Software, La Jolla, CA, USA), for statistical analysis. The data was expressed as mean ± S.D. and compared with One-way ANOVA. All pairs of columns were compared with Newman-Keul's test. Differences were regarded as statistically significant at p < 0.05.

## Abbreviations

ALT: alanine aminotransferase
AST: aspartate aminotransferase
BUN: urea nitrogen
Cr: creatinine
IgG: immunoglobulin G
Ip-Inj: intraperitoneal injection of IgG
Iv-Inj: intravenous injection of IgG
PWBC: peripheral white blood cells
Sub-Inj: subcutaneous injection of IgG
